# Experimental Gestational Diabetes Mellitus Induces Blunted Vasoconstriction and Functional Changes in the Rat Aorta

**DOI:** 10.1155/2014/329634

**Published:** 2014-12-28

**Authors:** Cecilia Tufiño, Cleva Villanueva-López, Maximiliano Ibarra-Barajas, Ismael Bracho-Valdés, Rosa Amalia Bobadilla-Lugo

**Affiliations:** ^1^Sección de Estudios de Posgrado e Investigación, Escuela Superior de Medicina, Instituto Politécnico Nacional, Plan de San Luis y Díaz Mirón, Colonia Santo Tomás, 11340 DF, Mexico; ^2^Unidad de Biomedicina, Facultad de Estudios Superiores Iztacala, Universidad Nacional Autónoma de México, Avenida De los Barrios 1, Los Reyes Iztacala, 54090 Tlalnepantla, Mexico

## Abstract

Diabetic conditions increase vascular reactivity to angiotensin II in several studies but there are scarce reports on cardiovascular effects of hypercaloric diet (HD) induced gestational diabetes mellitus (GDM), so the objective of this work was to determine the effects of HD induced GDM on vascular responses. Angiotensin II as well as phenylephrine induced vascular contraction was tested in isolated aorta rings with and without endothelium from rats fed for 7 weeks (4 before and 3 weeks during pregnancy) with standard (SD) or hypercaloric (HD) diet. Also, protein expression of AT_1_R, AT_2_R, COX-1, COX-2, NOS-1, and NOS-3 and plasma glucose, insulin, and angiotensin II levels were measured. GDM impaired vasoconstrictor response (*P* < 0.05 versus SD) in intact (e+) but not in endothelium-free (e−) vessels. Losartan reduced GDM but not SD e− vasoconstriction (*P* < 0.01 versus SD). AT_1_R, AT_2_R, and COX-1 and COX-2 protein expression were significantly increased in GDM vessels (*P* < 0.05 versus SD). Results suggest an increased participation of endothelium vasodilator mediators, probably prostaglandins, as well as of AT_2_ vasodilator receptors as a compensatory mechanism for vasoconstrictor changes generated by experimental GDM. Considering the short term of rat pregnancy findings can reflect early stage GDM adaptations.

## 1. Introduction

Approximately 7% of all pregnancies are complicated by gestational diabetes mellitus (GDM), a health problem that has recently been propelled by climbing obesity rates [1]. Maternal obesity commonly complicates pregnancies with GDM, T2DM, and even T1DM and independently increases the risk of adverse pregnancy outcomes [2].

Gestational diabetes mellitus (GDM) is defined by American Diabetes Association as any degree of glucose intolerance with onset or first recognition during pregnancy [3]. Women with GDM are at increased risk for the development of complications such as macrosomic product, preeclampsia [4], and diabetes, usually type 2, after pregnancy [5].

Both obesity and overweight are conditions associated with a decreased insulin sensitivity [6] and have been identified as the main risk factors for GDM [7]. In this sense, insulin resistance (IR) is known to be a key factor for vascular complications such as endothelial dysfunction and impaired vascular relaxation.

In turn, obesity induced cardiovascular and metabolic changes have been widely studied in animal models using high fat [8, 9] or fructose diet intake [10, 11]. Nevertheless, reports about the cardiovascular impact of hypercaloric diet in female rodents [12] and GDM models are scarce [13]. In this work, we developed an hypercaloric diet based model of GDM that alter glucose tolerance test (GTT) in pregnant rats without changing basal blood glucose levels, resembling the features of human obesity associated GDM.

On the other hand, the renin-angiotensin system (RAS) plays a critical role in the control of cardiovascular and renal functions [14] and all components of the RAS are present in blood vessels [15]. Indeed, angiotensin II exerts a potent role in the control of cardiovascular homeostasis through specific receptors, traditionally AT_1_R and AT_2_R. AT_1_R has demonstrated a crucial role in the diabetes/obesity enhanced response to angiotensin II [10] as well as in the pathogenesis of diabetic vascular dysfunction [16] and clinically on the basis of the therapeutic ability of angiotensin converting enzyme (ACE) inhibitors and AT_1_R blockers to decrease vascular complications in DM patients. On the other hand, potential counter regulatory vasodilator properties have been attributed to AT_2_R [17] and to other components of RAS such as ACE2-angiotensin 1–7 [15], which have shown an increased expression [18–20] in diabetic conditions which have been correlated with vasoprotective effects.

Additionally, there is evidence of changes in angiotensin II crosstalk between *α*1-adrenoceptor and angiotensin AT_1_ receptors [21] as an early damage indicator of metabolic alterations [9].

Considering aorta angiotensin II response has been used as a surrogate measure of large artery disease [9], in this work, we, therefore, intend to study the effect of GDM on vascular function, considering (1) if GDM increases Ang II induced vasoconstriction (2) if GDM changes the interaction between adrenergic alpha-1 and Ang II AT_1_ receptors in this vessel and (3) The participation of NOS and COX in such changes in the rat aorta.

## 2. Methods

### 2.1. Animals

12-week-old female Wistar rats weighing 250 ± 15 g were used. Animals were kept in 12-hour light/dark cycle and controlled humidity, with free access to food and water. All procedures were approved by the Official Mexican Norm (NOM-062-ZOO-1969) for the Animal Handling and were approved by the local Ethical and Research Committees of our institution ESM-IPN.

### 2.2. Diet-Induced Obesity

Rats had free access to a standard diet (SD) rat chow (3.1 kcal/g) or to a high-calorie diet (HD) (6.3 kcal/g) over 7 weeks. HD was prepared with 33% ground commercial rat chow; 33% full fat sweetened condensed milk (Nestle); 7% sucrose; and 27% water [13]. The diets and the water were provided ad libitum. Weight was recorded weekly. At the end of this period glucose tolerance test and plasma levels of insulin and angiotensin II were determined and the aortas excised.

### 2.3. Gestational Diabetes Mellitus

One group of animals was mated with male rats at the end of the 4th week of either HD or SD. Day 1 of pregnancy was considered 48 hours after mating (error margin ± 24 hours). Rats continued the diet for the average period of pregnancy (3 weeks).

### 2.4. Records in Whole Animal

Measurement of blood pressure was performed by indirect tail cuff plethysmography method (Letica 5007 PanLab, Barcelona). Rats were subjected to previous training for 3 days. Measurements were obtained at the beginning of the experiment and at the end of weeks 4 and 7. Procedure consisted in placing rats into appropriate traps inside a room free from noise and light, previously warmed to 32°C. Systolic blood pressure was determined as the mean value after 3 consecutive successful measurements.

A drop of blood obtained from the tail tip was used to determine blood glucose levels with an Accu-Chek Advantage glucose meter (Roche Diagnostics, Basel, Switzerland).

### 2.5. Glucose Tolerance Test

Morning glucose was measured before (min 0) and after 5, 10, 15, 30, 45, 60, 90, and 120 min, an intraperitoneal bolus of 1 g/kg glucose. Glucose tolerance was determined by calculating the area under the curve from min 5 to 120 values and given in arbitrary units (AU) Prism graph.

### 2.6. Studies in Isolated Organ

Under ether anesthesia, animals were sacrificed and the thoracic aorta was excised and cleaned from surrounding connective tissue. The isolated arteries were cut into 6 rings (3-4 mm long). Endothelium was removed from 3 of them, and each ring was placed in tissue chambers filled with 10 mL Krebs-Henseleit solution of the following composition (mM): NaCl 118, KCl 4.8, CaCl_2_ 2.5 MgSO_4_ 1.2, KH_2_PO_4_ 1.2, NaHCO_3_ 25, glucose 11.7, EDTA 0.026, maintained at 37°C, pH 7.4, and bubbled with 95% O_2_ containing 5% CO_2_. Rings were mounted on two Nikrom hooks in order to fix them to the bottom of the chamber and to a 50G-TSD125C force transducer connected to a general purpose amplifier DA100C, in turn coupled to a data acquisition system MP100 (Biopac System Inc., Santa Barbara, CA, USA). Vessels were given 3 g of initial tension and were prestimulated three times with phenylephrine (Phe) (1 × 10^−6^ M). The third time constriction was allowed to plateau and rings were exposed to acetylcholine (Ach) (10^−6^ M) to assess the presence of endothelium. Rings were considered to have endothelium if relaxation was ≥80%.

Graphs were constructed using the percentage of contraction respect to KCl maximal effect (100%).

### 2.7. Determination of Proteins by Western Blot

Protein expression of eNOS, iNOS, COX-1, and COX-2 enzymes and of AT_1_ and AT_2_ receptors was determined through Western Blot. Cleaned vessels from the four experimental groups (*n* = 4 per group) were homogenized in RIPA solution containing a mixture of protease inhibitors at low speed (between 10 000 y 15 000 rpm during 15 seconds for each pulse) followed by 10000 rpm for 10 min at 4°C centrifugation. Protein concentration was determined with the Lowry method. After b-mercaptoethanol (100°C for 10 min) treatment, equal amounts of protein (50 mg) were loaded on a 10% and 5% SDS-PAGE. They were subjected to electrophoresis (MiniPROTEAN) 25 min to 80 volts and 1.25 min to 120 volts and transferred to polyvinylidene fluoride membranes for 1 h at 15 V, using a semidry trans-blot (Bio-Rad Laboratories, Hercules, CA, USA). Membranes were blocked 2 h at room temperature in 5% low-fat milk washing solution. Then, membranes were incubated with goat polyclonal antibody against AT_1_R, AT_2_R, COX-1, COX-2, actin, or rabbit polyclonal antibody against iNOS and eNOS diluted 1 : 200, 1 : 400, and 1 : 1000, in washing solution at 4°C overnight.

Membranes were then washed five times, incubated with rabbit anti-goat or goat anti-rabbit horseradish peroxidase-conjugated second antibody 1 : 10000 for 2 h at room temperature and washed extensively. Membranes were incubated with chemiluminescence blotting substrate (Western Blotting Luminol Reagent, Santa Cruz Biotechnology, CA, USA) according to the manufacturer's protocol and exposed to film that was immediately developed. The film was scanned and band intensity was measured by computer analysis using gels densitometer BioSens SC 645 and was normalized with actin intensity (control protein).

### 2.8. Blood Sampling

Blood samples were obtained via cardiac puncture. Samples were stored at 4°C in Eppendorf tubes containing heparin, and centrifuged right after at 1500 rpm, 4°C, for 15 min. 1 mL serum was removed and 100 mL of protease inhibitors mixture was added. Immediately, the serum was frozen at −70°C for analysis of plasma peptide C and angiotensin II concentrations.

### 2.9. Determination of Plasma Angiotensin II and Peptide C

The determination of plasma levels of angiotensin II and peptide C, as a measure of insulin concentration, was conducted by ELISA kit for angiotensin II (Angiotensin II EIA kit, Cayman) or ELISA kit for peptide C (Human C-peptide ELISA, Millipore) following the manufacturer's recommendations.

### 2.10. Analysis and Statistics

In isolated organ experiments, each experimental group included 5-6 animals. Data are expressed as the mean ± SEM. pD2 (−Log EC_50_) and *E*_max⁡_ values were obtained by nonlinear regression analysis from concentration-response curves. Statistical evaluation of the data, when comparing each point of concentration response curve, was carried out by two-way ANOVA, with Bonferroni test for comparison of means. pD2 (−Log EC_50_) and *E*_max⁡_ values were compared by using unpaired Student's *t*-test.

For the Western Blot, values are expressed as arbitrary units that result from the coefficient AT_1_ or any/actin. They are the mean ± SEM of four experiments and were analyzed using unpaired Student's *t*-test.

In all comparisons, values of *P* < 0.05 were considered to indicate significant differences between the means.

## 3. Results

### 3.1. Body Weight and Determination of Blood Levels

**Figure 1:**
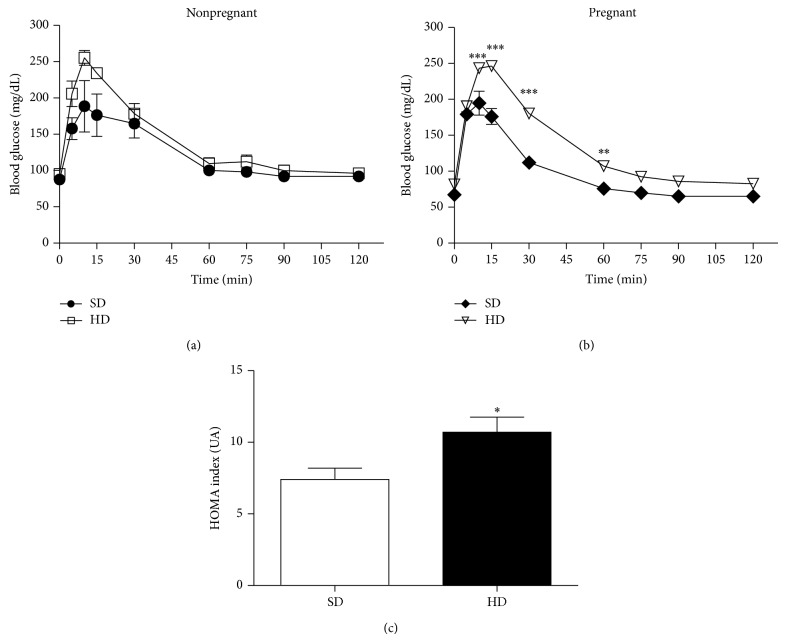
Intraperitoneal glucose tolerance test (IPGTT). Glucose concentrations during 120 min in (a) nonpregnant rats fed standard diet, SD (●), or hypercaloric diet, HD (□), and (b) pregnant rats fed standard diet, SD (♦), or hypercaloric diet, HD (▿). (c) HOMA index from pregnant rats (days 19–21) standard diet, SD (white), and hypercaloric diet, HD (black). ^*^ *P* < 0.05 versus SD, ^**^ *P* < 0.01 versus SD, and ^***^ *P* < 0.001 versus SD.

Four weeks of HD intake increased the rat body weight by 30.9% compared with 9.6% for rats on SD. This difference was even more evident at the end of the third week of pregnancy (125.62 ± 5.75 g versus 67.11 ± 4.23 g, *P* < 0.05) HD versus SD, respectively. Plasma glucose levels after 7 weeks of HD did not change with respect to SD. However, insulin (1.96 ± 0.2 ng/mL versus 1.23 ± 0.08 ng/mL, *P* < 0.05, HD versus SD, resp.) and angiotensin II (119.9 ± 5.36 ng/mL versus 101.9 ± 6.62 ng/mL, *P* < 0.05, HD versus SD, resp.) concentrations were significantly increased in HD pregnant rats (Figure 1), as well as HOMA index (10.70 ± 1.05 versus 7.40 ± 0.79, HD versus SD, resp.) (Figure 1(c)).

### 3.2. Blood Pressure

Blood pressure did not change in pregnant rats HD compared with SD fed (119 ± 3.52 mmHg versus 114.3 ± 2.65 mmHg HD and SD, resp.) (Table 1).

### 3.3. Glucose Tolerance

**Figure 2:**
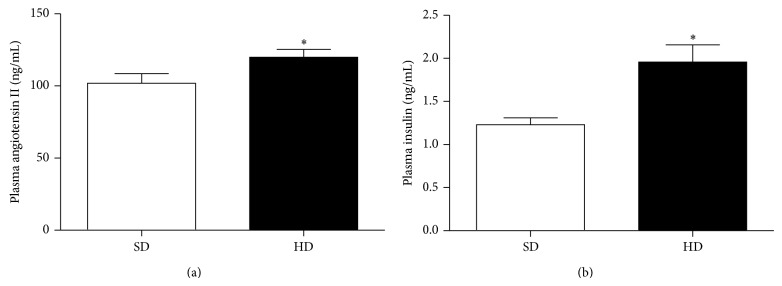
(a) Plasma angiotensin II levels and (b) plasma insulin levels from pregnant rats (days 19–21) in standard diet, SD (white), and hypercaloric diet, HD (black). Results are mean ± SEM of four experiments. ^*^ *P* < 0.05 versus SD.

In order to determine whether insulin resistance was produced by HD, a glucose tolerance test (GTT) was conducted at the end of the third week of pregnancy (end on the seventh week of diet). GTT was clearly impaired in HD compared to SD animals (1585 ± 21 versus 1151 ± 20.1 AU, ^*^ *P* < 0.05, HD and SD, resp.) (Figures 2(a) and 2(b)). The altered GTT in pregnant rats HD fed is a required feature for a GDM experimental model.

### 3.4. Isolated Organ Studies

In order to evaluate the smooth muscle conditions, aortic rings both with and without endothelium were challenged with KCl 80 mM. No differences were found between groups (3.58 ± 0.29 g versus 3.15 ± 0.21 g e+ HD versus SD, resp.) and without endothelium (3.46 ± 0.28 g versus 3.18 ± 0.31 g e− HD versus SD, resp.) (data not shown), suggesting that HD did not affect the contractile machinery of the vessel.

### 3.5. Relaxation: Response to Acetylcholine

To evaluate the role of HD in endothelium-dependent relaxation, acetylcholine (1 × 10^−6^ M) response was evaluated on intact aorta rings precontracted with Phe (1 × 10^−6^ M). Percentage of relaxation was increased in HD compared to SD aortas (97.13 ± 2.84% versus 84.97 ± 2.3%, *P* < 0.05, resp.) (data not shown).

### 3.6. Vasoconstriction: Response to Angiotensin II

**Figure 3:**
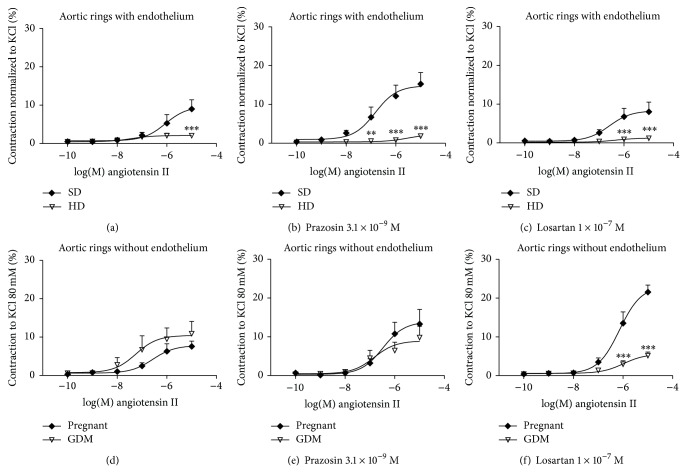
Contractions induced with angiotensin II in isolated intact thoracic aortic rings from pregnant rats fed standard diet, SD (♦), or hypercaloric diet, HD (▿). Graphs were constructed using the percentage of contraction respect to KCl maximal effect (100%). (a) Vessels without antagonists, (b) pretreated with prazosin (3.1 × 10^−9^ M) or (c) losartan (1 × 10^−7^ M). Contractions induced with angiotensin II in isolated thoracic aortic rings endothelium-denuded from pregnant rats fed standard diet, SD (♦), or hypercaloric diet, HD (▿). (d) Vessels without antagonists, (e) pretreated with prazosin (3.1 × 10^−9^ M) or (f) losartan (1 × 10^−7^ M). Dates are the mean ± SEM of 4–7 experiments. ^*^ *P* < 0.05 versus SD, ^**^ *P* < 0.01 versus SD, and ^***^ *P* < 0.001 versus SD.

Ang II concentration response curves to angiotensin II (10^−10^–10^−5^ M) were ran in aorta rings both with and without endothelium (*n* = 5) in both experimental groups, in the presence of prazosin 3.1 × 10^−9^ M, and losartan 1 × 10^−7^ M (Figure 3).

#### 3.6.1. Endothelium Intact

GDM decreased aorta contraction to Ang II (*E*_max⁡_  2.09 ± 0.47% versus 9.66 ± 1.7%, *P* < 0.05, HD and SD, resp.) (Table 2). Aorta ring incubation with prazosin or losartan did not change SD response but response of HD vessels remained decreased (Table 2).

#### 3.6.2. Endothelium-Denuded

Contraction to Ang II was restored to control levels in endothelium-free HD vessels (*E*_max⁡_  10.44 ± 1.81% versus 7.79 ± 0.99%, HD versus SD resp., n.s.d.) (Table 2), and also there was no difference between SD and HD groups in the presence of prazosin (*E*_max⁡_  8.95 ± 1.45% versus 13.88 ± 1.99% n.s.d. HD and SD, resp.) (Table 2). Interestingly, incubation with losartan significantly reduced the response to angiotensin II in HD vessels (*E*_max⁡_  5.6 ± 0.81% versus 22.02 ± 1.7%, *P* < 0.01, HD and SD, resp.).

### 3.7. Vasoconstriction: Response to Phenylephrine

In order to determine if the effect of GDM was specific for angiotensin II, the response to the alpha agonist Phe (10^−10^–10^−4^ M) in the absence or presence of prazosin 3.1 × 10^−9^ M or losartan 1 × 10^−7^ M was tested in both experimental groups.

#### 3.7.1. Endothelium Intact

**Figure 4:**
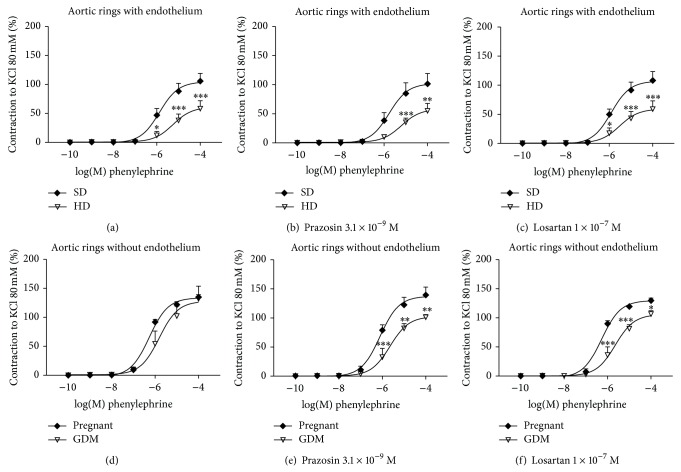
Contractions induced with phenylephrine in isolated intact thoracic aortic rings from pregnant rats fed standard diet, SD (♦), or hypercaloric diet, HD (▿). Graphs were constructed using the percentage of contraction respect to KCl maximal effect (100%). (a) Vessels without antagonists, (b) pretreated with prazosin (3.1 × 10^−9 ^M) or (c) losartan (1 × 10^−7^ M). Contractions induced with phenylephrine in isolated thoracic aortic rings endothelium-denuded from pregnant rats fed standard diet, SD (♦), or hypercaloric diet, HD (▿). (d) Vessels without antagonists, (e) pretreated with prazosin (3.1 × 10^−9^ M) or (f) losartan (1 × 10^−7^ M). Dates are the mean ± SEM of 4–7 experiments. ^*^ *P* < 0.05 versus SD, ^**^ *P* < 0.01 versus SD, and ^***^ *P* < 0.001 versus SD.

Similarly, HD reduced Phe induced contraction in vessels with endothelium (Figure 4) (*E*_max⁡_  60.9 ± 8.04% versus 104.6 ± 6.8%, *P* < 0.05, HD and SD, resp.) (Table 3). Aorta ring incubation with prazosin or losartan did not change SD response but response of HD vessels remained decreased (Table 3).

#### 3.7.2. Endothelium-Denuded

Contraction to Phe was unchanged in GDM vessels (*E*_max⁡_  127.9 ± 11.45% versus 133.8 ± 3.09% n.s.d. HD and SD, resp.) (Table 3). Phe induced contraction remained reduced in GDM vessels in the presence of prazosin (*E*_max⁡_  102.6 ± 6.1% versus 137.4 ± 6.2%, *P* < 0.01, HD and SD, resp.) (Table 3). Interestingly, and supporting cross-talk hypothesis, incubation with losartan significantly reduced the response to Phe (*E*_max⁡_  105.9 ± 5.7% versus 129.8 ± 3.23%, *P* < 0.05, HD and SD, resp.).

### 3.8. Receptors and Enzymatic Determination by Western Blot

#### 3.8.1. ATR Expression

**Figure 5:**
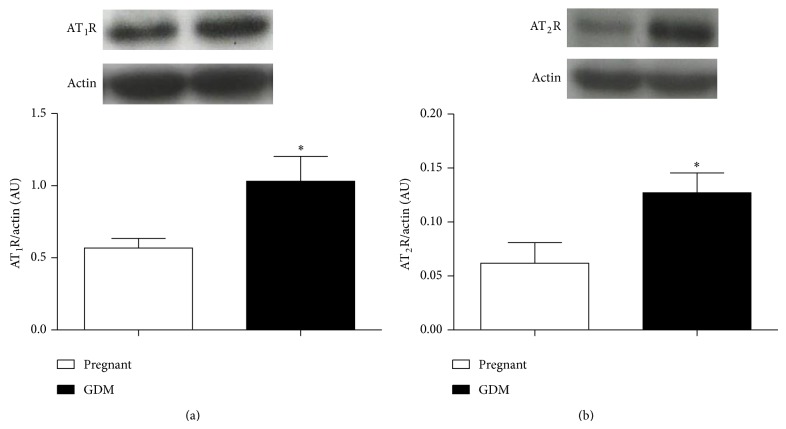
AT_1_ (a) and AT_2_ (b) receptor in thoracic aorta from pregnant rats fed standard diet, SD (□), and hypercaloric diet, HD (■). Data are the mean ± SEM of four rats. ^*^ *P* < 0.05 versus SD.

When studying the aorta protein expression of the receptors for Ang II and AT_1_ and AT_2_, the Western Blot analysis showed an increased expression (AU) for both receptors in HD compared to the SD rats (*P* < 0.05) (Figure 5).

#### 3.8.2. COX-1, COX-2, iNOS, and eNOS Expression

**Figure 6:**
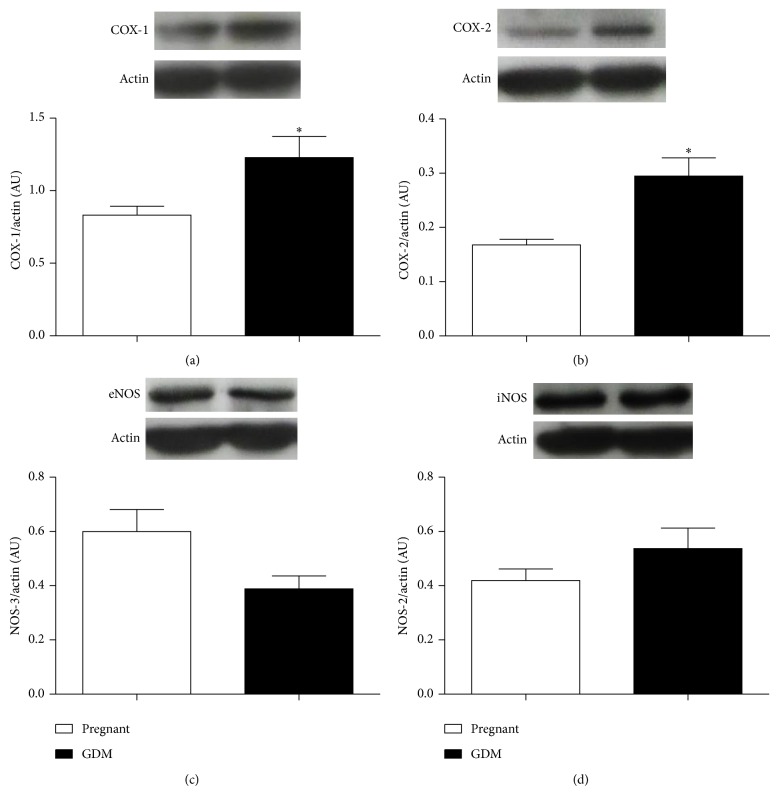
(a) Cyclooxygenase-1, (b) cyclooxygenase-2, (c) endothelial nitric oxide synthase, and (d) induced nitric oxide synthase in thoracic aorta from pregnant rats fed standard diet, SD (□), and hypercaloric diet, HD (■). Data are the mean ± SEM of four rats. ^*^ *P* < 0.05 versus pregnant.

Also, a significant increase in both COX-1 and COX-2 enzymes protein expression (AU) was found in vessels from HD compared with SD (*P* < 0.05) (Figures 6(a) and 6(b)).

No differences in expression of iNOS and eNOS (AU) were found between the two experimental groups (Figures 6(c) and 6(d)).

## 4. Discussion

This study examined whether HD induced GDM modulates changes in vascular reactivity. Results demonstrate GDM decreased vasoconstriction by Ang II or Phe in an endothelium dependent way. These changes were associated with glucose intolerance: increased insulin and HOMA index as well as Ang II plasmatic levels.

In the present work, basal blood glucose was unchanged by HD in either group but an abnormal glucose test was induced in HD pregnant rats, a result that supports the experimental model of GDM. Indeed, GDM is defined as a glucose tolerance disorder which is first diagnosed in pregnancy with oral glucose tolerance test (OGTT) [22]. In agreement, HD modifies GTT in pregnant but not in control rats [13]. In contrast, in similar models using male rodents, HD produced glucose intolerance [23]. Also, female rats did not develop hypertension or hyperinsulinemia upon fructose feeding except after ovariectomy [24] suggesting female condition protects against metabolic risk of glucose intolerance.

Also, Ang II has been proposed as an important mediator of hypercaloric diet [10, 25] and obesity [26] induced IR. So in the present work we tested the hypothesis that GDM condition increases Ang II induced vasoconstriction. Even when Ang II and insulin levels as well as AT_1_R and AT_2_R expression were found increased, and contrary to expectations, our main findings showed that GDM reduced the Ang II or Phe induced vasoconstriction. Interestingly, this effect was lost when endothelium was removed. Besides, HD did not increase systolic blood pressure in this experimental model of disease.

There are few animal models of GDM and this decreased response to vasoconstrictors is not a common finding in HD fed animals. Indeed, 4 weeks of hypercaloric diet enhanced Ang II-mediated aortic vasoconstriction of Sprague-Dawley rats [27] and enhanced coronary arteriolar Ang II response in dogs [28]. Moreover, in C57BL/6 mice, 15–30 weeks of hypercaloric diet increased aortic Ang II response linked to increased AT_1_R expression [29]. Also, an increased BP as well as vascular reactivity has been described in rodents with high fructose diet [11, 16]. Also, hypercaloric diet has demonstrated a rise in blood pressure [30] within a period of 4–8 weeks [31], although others found no differences [32, 33]. Specifically, in aortas from streptozotocin induced DMG rats, the response to Ang II was increased [34] and an impaired endothelial response in arteries from pregnant women [35] has also been described. Importantly, there are significant differences in the level of hyperglycemia with these reports that can explain our findings: average basal blood glucose levels from GDM rats were 88.1 ± 6.09 mg/dL in this work versus 452.5 ± 29.67 mg/dL in the former rat study. Besides, some reports suggest the development of endothelial dysfunction and elevated blood pressure in these models are dependent on the presence of testosterone [36]. Then, results suggest female rats show resistance for metabolic and cardiovascular impact of HD, particularly, during pregnancy. Nevertheless other factors such as diet duration/composition also must be considered to explain differences between models.

Indeed, estrogens have shown a protective effect against hypertension [37, 38] while testosterone favors an inverse effect [39]. Both male and female rats that were chronically treated with insulin exhibited impaired insulin sensitivity, which occurred to a greater degree in male rats [24]. Interestingly, only hyperinsulinemic male rats developed elevations in blood pressure [24].

On the other hand, whole renin angiotensin system (RAS) components are present in vascular tissue. Angiotensin II, the more extensively studied peptide of the system, stimulates AT_1_R (mainly associated with vasoconstriction) and AT_2_R (associated with vasodilation) but recently the role of other components of the system in vascular tone has also been considered. Angiotensin 1–7 (Ang 1–7) from Ang II by the angiotensin converting enzyme 2 (ACE2) and induces vasodilation [15]. Also, angiotensin IV derived from Ang I or Ang II produces vasodilation [40].

In the present work, we found that GDM rats showed an increased plasma level of Ang II, in agreement with fructose fed Sprague-Dawley rats [41] and other models [16, 42]. Increased Ang II levels can be related to the maintenance of a vicious cycle with insulin resistance [43].

The reduced responses of GDM aorta to Ang II and Phe suggest an increased participation of endothelium derived vasodilators. Besides, losartan decreased the response of endothelium denuded vessels suggesting a simultaneous increased AT_2_R participation. Then findings suggest an indirect “vasoprotective” effort of endothelium in face to hypercaloric diet induced vasoconstrictor changes such as increased AT_1_R and endothelin-1 expression. These vasoprotective effects are further supported by the increased Ach relaxation observed in the GDM group.

In accordance, there is an upregulation of AT_2_ receptors in rat thoracic aorta under conditions associated with vascular tissue damage, such as diabetes and hypertension [20, 44], and Ang II produced a concentration-dependent relaxation in endothelium-intact and endothelium-denuded rat thoracic aorta in the presence, but not in the absence, of AT_1_ selective antagonists (losartan or valsartan) [20].

Then, suppression of Ang II-mediated responses in GDM may also be linked to a local rise in AT_2_R [20]. Also, Ang 1–7 and its receptor “mas”, can also have a role, considering these RAS system factors increase after HD [15, 18, 45]. Moreover, it has been found that ACE2 activation improves endothelial function [46] and is regulated by a high-fat diet [33] and high sucrose intake in rats [45]. ACE levels were not measured in this work, but the hypothesis of increased formation of angiotensin 1–7 to explain the blunted contraction elicited by GDM aortas cannot be discarded. Further research will examine whether HD-mediated enhancement of aortic endothelial function in GDM rats is mediated by the activation of ACE.

Participation of other vasodilator mediators must not be ignored: both the AT_1_R [47] and AT_2_R [48] activate NOS. Also both in obesity [49, 50] and in diabetes [51], an increase in the expression of NOS-2 and of COX-2 has been reported. In a separate study, aortic tissue from fructose-fed rats had increased expression of inducible COX-2 [52].

We found protein NOS expression was unchanged in this work but GDM vessels showed increased levels of both COX-1 and COX-2, suggesting an increased participation of vasodilator prostaglandins probably, as angiotensin 1–7 mediators [53]. Separate studies have demonstrated altered vascular release of prostaglandins in arteries from fructose-fed rats. Following long-term fructose feeding, aortas released lower levels, whereas mesenteric beds released greater amounts of PGI2 [54]. Then, it is possible that GDM aortas can release an increased amount of this endothelium derived prostanoid.

Furthermore, cross talk between *α*1-adrenoceptors and angiotensin AT_1_ receptors in the smooth muscle of rabbit aorta [21] is modified by hypercholesterolemia [9] and has been proposed as a mechanism for the onset and progression of chronic vascular diseases. So, in the present work we also tested the hypothesis that GDM conditions modify the response specifically to angiotensin II but may change *α*1-adrenoceptor and angiotensin AT_1_ receptor interaction compared to controls.

Interestingly, endothelium denuded GDM aortas maintained a blunted response to the alpha adrenergic agonist Phe in the presence of either prazosin or losartan, suggesting vascular changes are not Ang II specific. Besides, results unmask a cross-talk between alpha adrenergic and AT_1_ receptors. Evidence of alpha adrenergic and Ang II cross-talk has been described in rabbit aorta [21]. And also prazosin has been shown to antagonize AT_1_R in the same vessel [55] as well as to improve insulin resistance [56]. On the other hand, AT_1_R antagonism modifies the mediation of catecholamines in the renal constrictor response to angiotensin [57].

These data highlight the susceptibility of cardiovascular disease via changes in receptor number/sensitivity in GDM rats compared with normal pregnant animals. Intriguingly, GDM is associated with hypertensive disorders of pregnancy in humans [58], so the phenomena described in the present study in rats, a species with an average pregnancy period of 3 weeks, can reflect vasorelaxant mechanisms at early stages that are surmounted in the 40-week-long human pregnancy.

Taken together, the present data are compatible with the notion that diet induced provasoconstrictor damage in GDM rat aortas is compensated by vasodilator activity mainly endothelium dependent, represented by an increased participation of vasodilator members of RAS such as AT_2_R; further study is needed for clarifying the participation of other mediators, as well as the response of resistance vessels and the long term effects of the changes observed.

## Figures and Tables

**Table 1:** Blood pressure from pregnant rats fed standard diet (SD) and hypercaloric diet (HD) taken at the beginning of the protocol and the four and seven weeks.

SD	HD	
Baseline (mmHg)	132.8 ± 2.518	136.8 ± 4.893
Week 4 (mmHg)	131.6 ± 1.939	139.5 ± 3.014
Week 7 (mmHg)	114.3 ± 2.654	119 ± 3.521

**Table 2:** Angiotensin II *E*_max⁡_ (%) and pD2 (log⁡M) of aortic rings from pregnant rats fed SD and HD with and without endothelium.

	Endothelium-intact aortic rings			
	SD		HD	
	*E*_max⁡_ (%)	pD2 (log⁡M)	*E*_max⁡_ (%)	pD2 (log⁡M)
Ang II	9,658 ± 1,733	6,059 ± 0,3473	2,093 ± 0,4685^&&^	7,434 ± 0,8589
Ang II/prazosin	14,84 ± 1,673	6,828 ± 0,2918	2,301 ± 0,5973^&&&^	5,517 ± 0,4347^&^
Ang II/losartan	8,293 ± 1,279	6,595 ± 0,3825	1,265 ± 0,2893^&&^	6,328 ± 0,5651

	Endothelium-denuded aortic rings			
	SD		HD	
	*E*_max⁡_ (%)	pD2 (log⁡M)	*E*_max⁡_ (%)	pD2 (log⁡M)

Ang II	7,794 ± 0,9906	6,571 ± 0,321	10,44 ± 1,81	7,247 ± 0,5114
Ang II/prazosin	13,88 ± 1,99^*^	6,484 ± 0,3324	8,956 ± 1,454	6,829 ± 0,4150
Ang II/losartan	23,02 ± 1,756^***^	6,145 ± 0,1467	5,593 ± 0,8142^&&&^	5,984 ± 0,2810

Values are mean ± SEM for at least five experiments. ^&^ *P* < 0.05 versus SD; ^&&^ *P* < 0.01 versus SD; ^&&&^ *P* < 0.001 versus SD. ^*^ *P* < 0.05 versus control. ^***^ *P* < 0.001 versus control.

**Table 3:** Phenylephrine *E*_max⁡_ (%) and pD2 (log⁡M) of aortic rings from pregnant rats fed SD and HD with and without endothelium.

	Endothelium-intact aortic rings			
	SD		HD	
	*E*_max⁡_ (%)	pD2 (log⁡M)	*E*_max⁡_ (%)	pD2 (log⁡M)
Phenylephrine	104,6 ± 6,817	5,874 ± 0,1533	60,9 ± 8,037^&&^	5,252 ± 0,2627
Phen/Prazosin	102,1 ± 9,075	5,763 ± 0,2074	58,12 ± 6,677^&&^	5,204 ± 0,2239
Phen/Losartan	107,2 ± 6,777	5,906 ± 0,1488	59,15 ± 7,547^&&^	5,523 ± 0,2896

	Endothelium-denuded aortic rings			
	SD		HD	
	*E*_max⁡_ (%)	pD2 (log⁡M)	*E*_max⁡_ (%)	pD2 (log⁡M)

Phenylephrine	133,8 ± 3,092	6,265 ± 0,0608	127,9 ± 11,45	5,808 ± 0,2126
Phen/Prazosin	137,4 ± 6,2	6,102 ± 0,1124	102,6 ± 6,131^&&^	5,644 ± 0,1385
Phen/Losartan	129,8 ± 3,239	6,269 ± 0,0654	105,9 ± 5,799^&^	5,644 ± 0,1268

Values are mean ± SEM for at least five experiments.

^&^ *P* < 0.05 versus SD; ^&&^ *P* < 0.01 versus SD.
